# Lessons for the Clinical Nephrologist: Nephrotic syndrome associated with refractory *Giardia duodenalis* infection in a patient with acquired B cell depletion

**DOI:** 10.1007/s40620-023-01601-3

**Published:** 2023-03-17

**Authors:** Michael Eder, Lisabeth Pimenov, Georg A. Böhmig, Barbara Kornek, Lisa Göschl, Gregor Bond, Matthias G. Vossen, Winfried F. Pickl, Monika Breuer, Irene Görzer, Nicolas Kozakowski, Hermann Laferl, Stefan Winkler

**Affiliations:** 1 Division of Nephrology and Dialysis, Department of Medicine III, Medical University of Vienna, Vienna, Austria; 2 Institute of Immunology, Center for Pathophysiology, Infectiology and Immunology, Medical University of Vienna, Vienna, Austria; 3 Department of Neurology, Medical University of Vienna, Vienna, Austria; 4 Division of Rheumatology, Department of Internal Medicine III, Medical University of Vienna, Vienna, Austria; 5 Division of Infectious Diseases and Tropical Medicine, Department of Internal Medicine I, Medical University of Vienna, Vienna, Austria; 6 Division of Clinical Virology, Department of Laboratory Medicine, Medical University of Vienna, Vienna, Austria; 7 Center for Virology, Medical University of Vienna, Vienna, Austria; 8 Department of Pathology, Medical University Vienna, Vienna, Austria; 9 Department of Medicine 4, Kaiser Franz Josef Hospital-Clinic Favoriten, Vienna, Austria

**Keywords:** Nephrotic syndrome, Minimal change disease, Giardia duodenalis

## The case

**Fig. 1:**
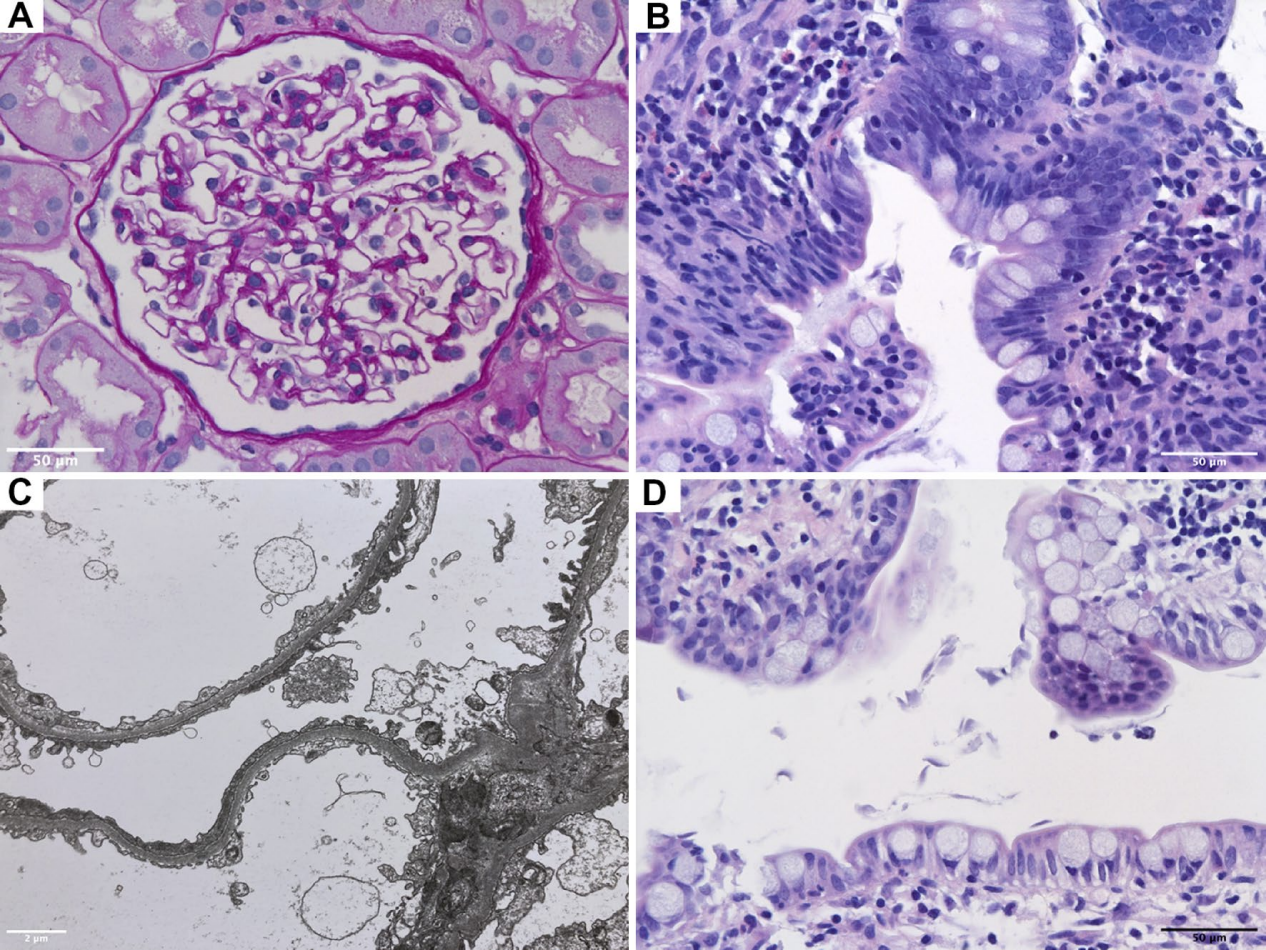
**A**, **C** Kidney biopsy: light microscopy with Periodic Acid-Schiff (PAS) stain showing regular kidney morphology (scale bar 50 µm). Electron microscopy showing segmental podocyte foot process effacement (30%) without electron dense deposits (scale bar 2 µm). **B**, **D** HE demonstrating terminal ileum with chronic mild ileitis and pear- or sickle-shaped micro-organisms consistent with trophozoites of Giardia Lamblia (scale bar 50 µm)

**Fig. 2:**
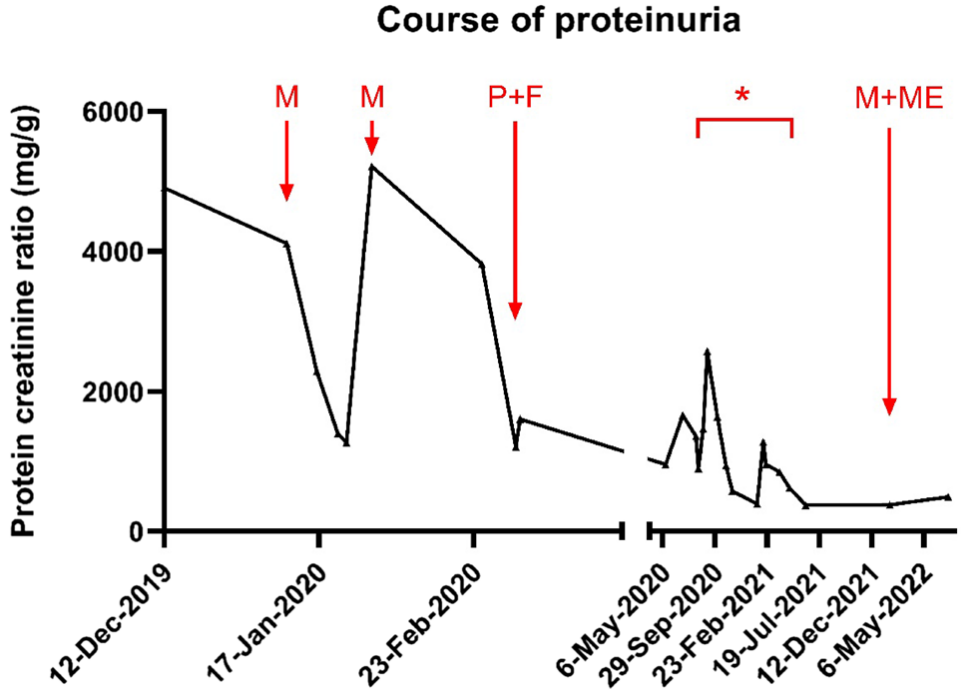
Timeline demonstrating the effects of different antimicrobial regimens on the course of the patient’s protein/creatinine ratio. After two initial treatment cycles with metronidazole, the urinary protein loss significantly decreased. *indicates the time period with different combination regimens including metronidazole, albendazole and paromomycin (see text above). Tinidazole and immunoglobulin substitution were recommended by the infectious disease board, however the necessary import of tinidazole was not supported by the patient’s health insurance. In February 2020, the patient additionally received Pivmecillinam and Nitrofurantoin due to a urinary tract infection with *Escherichia coli*. *F* nitrofurantoin, *M* metronidazole, *ME* mepacrine, *P* pivmecillinam

A 32-year-old female Caucasian patient presented to the emergency department in January 2020 with diarrhea, fever, cough and progressing leg edema, after returning from Turkey. Patient history revealed relapsing remitting multiple sclerosis (MS) since 2003 with various immunosuppressive treatments. In 2013 she received rituximab (2 × 375 mg/m^2^ body surface, two weeks apart), and no further MS relapses recurred thereafter (EDSS 2.0). Initial chest CT scans showed extensive bilateral lung infiltrations and pleural effusions, while laboratory results revealed microcytic, hypochromic anemia (Hb 9.9 g/dL), elevated acute phase parameters (leukocytosis 16.6 G/L, C-reactive protein 307.7 mg/L), hypoalbuminemia (1.84 g/dL) and proteinuria (spot urine protein/creatinine ratio 1777 mg/g, normal serum creatinine). Empiric treatment with cefuroxime, azithromycin and loop diuretics improved most symptoms, except diarrhea. Stool testing indicated infection with *Giardia duodenalis*. Oral metronidazole transiently resolved the symptoms, however diarrhea reoccurred and persistence of *Giardia duodenalis* was verified in stool samples by PCR. Proteinuria further increased (protein/creatinine ratio 5200 mg/g, normal serum creatinine). A subsequent kidney biopsy revealed mild subacute tubular injury but otherwise regular parenchyma (light microscopy, Fig. 1A) and segmental podocyte foot effacement (electron microscopy, Fig. 1C); minimal change nephropathy was diagnosed. Neither glucocorticoids nor angiotensin-converting enzyme inhibitors were given due to infection and hypotension, however, proteinuria improved upon sole antiparasitic treatment with two rounds of metronidazole (Fig. 2).

**Fig. 3:**
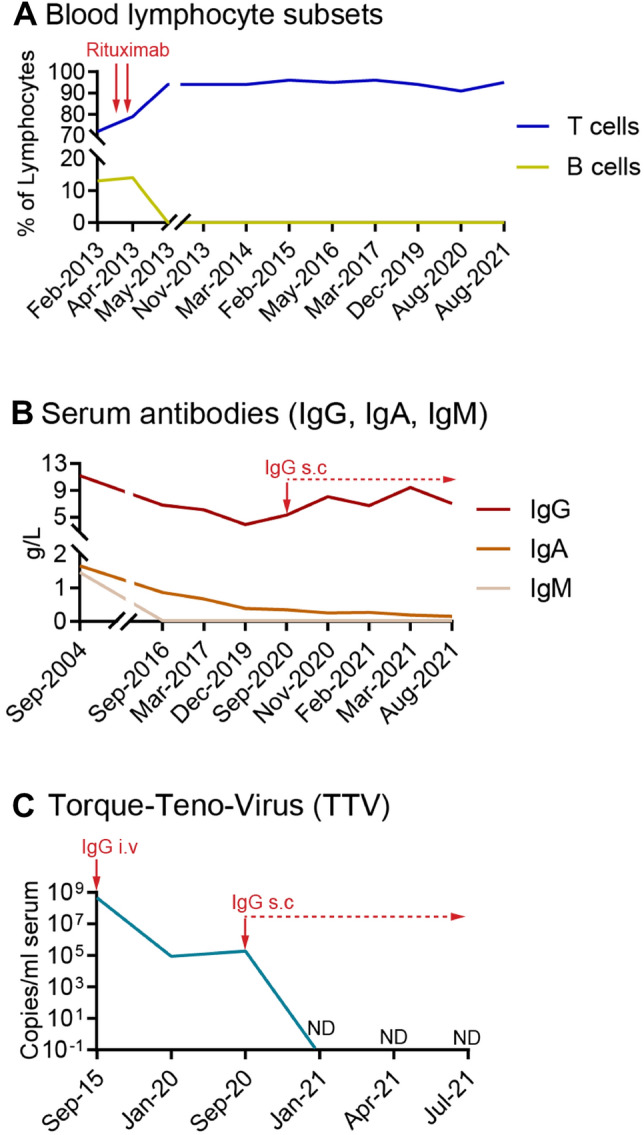
Immunological laboratory parameters over time. **A** Shown are the percentages of T cells (blue) and B cells (green) as percentages of blood lymphocytes over time. The application of two doses of rituximab treatment (red arrows) are indicated. **B** Shown are the serum levels of IgG (red), IgA (orange) and IgM (beige) over time. The patient received IVIG in the years 2014 and 2015 during her pregnancy (not shown) and later on as s.c. formulation (from 2020 ongoing, indicated by a red arrow). **C** Shown are Torque Teno virus copy numbers in serum over time, the time points/periods of IgG substitution are indicated by arrows and dashed line. *ND* non detectable

After one recurrence of diarrhea, successfully treated with metronidazole, the patient returned in May 2020 with persistent diarrhea. A subsequent gastro-colonoscopy confirmed giardiasis with ileitis but no signs of inflammatory bowel disease (Fig. 1B, D). Due to the recurrent, refractory infection, and consanguineous parental background, detailed testing for an immunodeficiency was initialized (Supplemental Table 1). Notably, reduced serum immunoglobulins (IgG 5.17 g/L, IgA 0.35 g/L, IgM < 0.52 g/L), and an absence of all circulating B cell subsets was found. B cell depletion had persisted since rituximab treatment in 2013 (Fig. 3A). Genetic testing revealed no cause for B cell depletion and no hereditary kidney disease.

Since a recommended treatment of tinidazole was not supported by the patient’s insurance, an alternative oral combination treatment with metronidazole and albendazole (14d), followed by paromomycin (14d) was initiated. Symptoms recurred after two months, therefore treatment was re-initiated with a repetitive albendazole and metronidazole combination, and while renal symptoms improved (Fig. 2), diarrhea persisted. In January 2022, the patient had COVID-19, and received sotrovimab and intravenous IgG. Refractory giardiasis was treated with quinacrine and metronidazole (5d) and finally, diarrhea disappeared. At last follow-up (July 2022), the patient was still asymptomatic.

## Lessons for the clinical nephrologist

In this report, we describe a young female patient with long-term B cell depletion and nephrotic syndrome associated with giardiasis. Our case provides a rare example of minimal change nephropathy secondary to *Giardia duodenalis* parasite infection in an immunodeficient patient with notable amelioration of renal symptoms after successful treatment of the refractory giardiasis, underlining the importance of careful interdisciplinary investigation of challenging patients.

## What caused refractory giardiasis in our patient?

Giardiasis is considered one of the most common protozoal infections worldwide. Transmission occurs through ingestion of contaminated water, food or by fecal–oral route. Despite global distribution and a considerable number of infections in developed countries (about 20,000 cases/year in the United States) it is a common cause of travelers’ diarrhea in individuals returning from settings with poor sanitation [1]. Refractory *Giardia duodenalis* infection has been connected to patients with common variable immunodeficiency (CVID) and especially B cell defects [2]. In our patient, we suspect that B cell depletion was the result of rituximab treatment for severe multiple sclerosis during adolescence, which led to the complete cessation of MS flares despite several other previous immunosuppressive regimens. Peripheral B cell repopulation times after rituximab treatment were reported to differ significantly depending on the primary autoimmune disease, with extremely prolonged repopulation intervals up to 149 months being observed in patients with underlying immunodeficiencies [3]. It has further been suggested that especially female patients are at risk of higher rituximab serum concentrations, possibly also contributing to prolonged B cell repopulation intervals [4]. It remains to be seen whether peripheral B cells will regenerate and proliferate again in our patient, who is now nine years post rituximab therapy. Another possible explanation for the refractory giardiasis in our patient is her consanguineous background (parents were cousins), with consanguinity often being associated with CVID. Although a primary immunodeficiency such as CVID was discussed in our patient, a clear diagnosis could not be made because immunoglobulins were detectable before rituximab administration. Levels of Torque Teno virus (TTV), a highly prevalent, non-pathogenic virus associated with degree of immunosuppression, are an emerging non-invasive method to estimate the intensity of individual immunosuppression [5]. Therefore, we retrospectively determined TTV levels in archived plasma samples from our patient, which revealed interesting results (Fig. 3C): the initially highly elevated TTV levels (10^8^ copies/ml) were measured when the patient gave birth to her third child in 2018 and were consistent with immunosuppressed patients. Five years later, despite persistent B cell depletion and severe immunoglobulin deficiency, TTV levels were comparable to healthy individuals (10^4^–10^5^). Finally, after immunoglobulin substitution, TTV disappeared and was undetectable in the further course.

## Can giardiasis trigger nephrotic syndrome?

Parasitic infections have been associated with all forms of kidney injuries, from asymptomatic urinary abnormalities to acute kidney injury, nephrotic syndrome and progressive chronic kidney disease. Despite its high prevalence, reports of *Giardia duodenalis-*triggered minimal change disease are rare. To the best of our knowledge, only two studies described giardiasis in the context of nephrotic syndrome in children and adults [6, 7]. Both studies relied on clinical associations, while the exact mechanisms remained unknown. In our case, both the initial trigger of nephrotic syndrome as well as the rapid improvement with antiparasitic treatment support the theory of giardiasis as the causative trigger for the patient´s kidney disease. The only segmental podocyte effacement may be explainable by the timing of biopsy. The highest level of proteinuria was measured approximately two weeks before the biopsy. During those two weeks the patient received anti-parasite treatment with metronidazole. Unfortunately, proteinuria levels were not available from the day of biopsy but it may be hypothesized that podocyte effacement might have been more advanced in an earlier biopsy.

## How should we treat refractory Giardiasis in immune-compromised patients?

Drug resistance of multiple antiparasitic drugs including metronidazole, albendazole and paromomycin have been described for *Giardia duodenalis* [8], but testing for drug resistance was not performed at our center. The applied antiparasitic regimen was congruent with treatment recommendations, and our case is also in line with reports describing that treatment combination in some immunocompromised patients was only successful with quinacrine [9], which was initially not available in Austria and had to be imported from the United Kingdom.

## Which lessons could be drawn from the case report?

In conclusion, our case report highlights some important clinical lessons. Although rarer in temperate climate zones, nephrotic-range proteinuria can be caused by giardiasis and physicians must be extra cautious before high-dose glucocorticoid treatment is initiated. Identification of refractory giardiasis should lead to screening for an underlying immunodeficiency. In such cases, a first-line treatment regimen including metronidazole and albendazole may not be effective and an earlier switch to alternative drugs may be justified. As such, quinacrine has repeatedly been reported as a successful rescue treatment. Although possibly beneficial for our patient´s neurological disease, prolonged B cell depletion may occur after rituximab treatment and such patients may show complications observed in individuals suffering from CVID. To date, the effect of immunoglobulin substitution on TTV levels is not known and needs to be further studied in detail. Once infection is controlled by anti-microbial treatment, proteinuria and nephrotic range symptoms may spontaneously decline. Therefore, in minimal change disease caused by parasites, immunosuppressive treatment may not be necessary but should rather be considered as potentially harmful.

## Supplementary Information

Below is the link to the electronic supplementary material.

Supplementary file1 (DOCX 107 kb)
