# Supplementary material for: Lessons for the Clinical Nephrologist: Nephrotic syndrome associated with refractory Giardia duodenalis infection in a patient with acquired B cell depletion
Source: J Nephrol. 2023 Mar 17;36(4):1197–201. doi: 10.1007/s40620-023-01601-3 (PMC10021039; doi:10.1007/s40620-023-01601-3)
Supplement: Supplementary file 1 — Supplementary file1 (DOCX 107 kb) [file 40620_2023_1601_MOESM1_ESM.docx]

Supplemental Table 1:

| Table 1 – April 2021 | | | | | | | |
| --- | --- | --- | --- | --- | --- | --- | --- |
| Serum immunoglobulin (Ig) levels | | | | | | | |
| Ig subtype | Serum levels (g/L) | | Normal range (g/L) | | | | |
| IgG | 5.17 | | 6.0-16.0 | | | | |
| IgA | 0.35 | | 0.8-3.0 | | | | |
| IgM | <0.52 | | 0.52-2.5 | | | | |
| Flow cytometric analysis FACS of blood | | | | | | | |
| Cell type | Subset | Lymphocyte phenotype | | Percentage of parent population (%) | Normal range (%) | Absolute cell numbers  (10^9^ cells/L) | Normal range (10^9^ cells/L) |
| Leukocytes |  |  | | - | - | 7.10 | 3.88-10.64 |
| Lymphocytes |  |  | | 35 | 27-34 | 2.49 | 1.0-2.8 |
| B cells | all | CD19/CD20+ | | 0 | 6-19 | 0 | 0.1-0.5 |
|  | IgM memory | CD19/CD20+IgD+ CD27+ | | 0 | 5-57 | 0 | 0.01-0.11 |
|  | Isotype-switched, memory | CD19+/CD20+IgD-CD27+ | | 0 | 7-39 | 0 | 0.01-0.08 |
|  | CD21 positive | CD19+/CD20+CD21+ | | 0 | 85-100 | 0 | 0.06-0.31 |
| T cells | all | CD3+ | | 95 | 55-83 | 2.36 | 0.7-2.1 |
|  | Naïve | CD3+CD45RA+ | | 37 | 16-49 | 0.87 | 0.16-0.61 |
|  | Memory | CD3+CD45RO+ | | 56 | 43-77 | 1.32 | 0.41-1.2 |
|  | CD4 | CD3+CD4+CD8- | | 61 | 28-57 | 1.52 | 0.3-1.4 |
|  | Naïve CD4+ | CD3+ CD4+CD8-CD45RA+ | | 46 | 26-42 | 0.7 | 0.08-0.55 |
|  | Memory CD4+ | CD3+ CD4+ CD8- CD45RO+ | | 40 | 19-77 | 0.94 | 0.22-0.97 |
|  | CD8 | CD3+ CD4- CD8+ | | 29 | 10-39 | 0.72 | 0.2-0.9 |
|  | Naïve CD8+ | CD3+ CD4-CD8+ CD45RA+ CD62L+ | | 62 | 45-61 | 0.45 | 0.06-0.59 |
|  | Memory CD8+ | CD3+CD4-CD8+CD45RO+ | | 30 | 8-29 | 0.71 | 0.06-0.62 |
|  | Double negative | CD3+ CD4-CD8- | | 0.9 | 0.5-2.4 | 0.02 | 0.01-0.04 |
| Granulocytes |  |  | | 52 | 50-75 | 3.69 | 2.02-8.22 |
| Monocytes |  |  | | 13 | 5-10 | 0.92 | 0.22-0.99 |
| Functional lymphocyte proliferation analyses | | | | | | | |
| Stimulation of lymphocytes for 4 or 7 days with: | | | | | | | |
| Control (medium) | | | | No proliferation | | | |
| Phytohemagglutinin PHA, anti-CD3 antibody, superantigen SEA, superantigen SEB, phorbol ester PMA, PMA/Ionomycin, Tetanus toxoid TT, tick-bourne encephalitis TBE | | | | Proliferation within age-matched normal limits | | | |
| Functional assessment of complement activation | | | | | | | |
| Classical complement pathway | | | | Activation within age-matched normal limits | | | |
| Alternative complement pathway | | | | Activation within age-matched normal limits | | | |
| Lectin complement pathway | | | | Activation within age-matched normal limits | | | |
| Inducible T cell costimulatory ICOS positivity with or without PHA stimulation | | | | | | | |
| ICOS + on unstimulated lymphocytes | | | | <0% | | | |
| ICOS + on lymphocytes stimulated with PHA for 48 hours | | | | Percentage within age-matched normal limits | | | |

**Supplemental table 1** – Summary of immunological work-up of the patient in April 2021. Normal range is defined by adult reference values, differences from the range in absolute numbers are marked by yellow background: Flow cytometric analysis (FACS) of the peripheral blood revealed a complete depletion of circulating B cells (0% CD19/CD20+). The depletion included B cell subsets such as the isotype-switched memory B cells (0% CD19+ IgD-CD27+), IgM memory B cells (0% CD19+ IgD+ CD27+) and as well CD21+ B cells (0% CD19+ CD21+). Since the beginning of rituximab therapy in 2013, B cell depletion persisted (through subsequent years, Figure 3B) Within the T cell compartment, we found an absolute and relative T lymphocytosis (95% of lymphocytes, 2.34x10^9^ cells/L, normal range: 55-83%, 0.7-2.1x10^9^ cells/L), with an absolute increase of the naïve CD4+ T cell fraction. Consistent with the B cell depletion, a relative increase of CD3+ T cells was observed since 2013, yet the absolute CD3+ T cells numbers were only elevated since the beginning of symptoms in December 2019. Functional analyses of lymphocyte proliferation with various mitogens (PHA, PMA, PMA/Ionomycin, Anti-CD3) and antigens (SEA, SEB, tetanus toxoid, FSME) showed proliferation within normal limits. Importantly, ICOS deficiency was not found upon stimulation with PHA. Functional assessment of complement activation via classical-, alternative-, and MBL pathways, was normal. Concerning the innate immune cells, FACS analyses showed normal granulocyte and monocyte numbers, as well as normal expression activation markers, with a singular increase of CD16 on monocytes. Especially, there was neither relative nor absolute increase of eosinophils at the time of Giardia diagnosis (only later a mild elevation during antiparasitic treatment was observed).

Supplemental Figure 1:


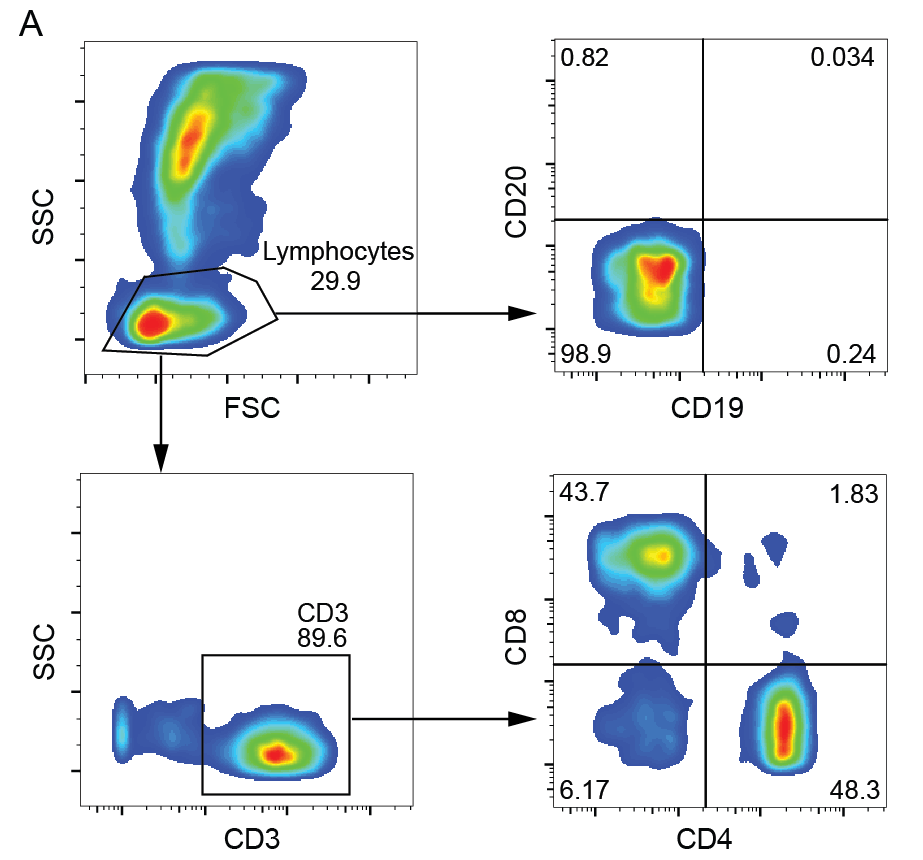


**Supplementary Figure 1: Dual parameter density plots of lymphocyte subsets of the patient in April 2021**. A) Lymphocytes are gated from the forward- and side scatter plot, and subsequently gated into CD19/CD20 positive B cells, or CD3+ T cells, which are further subdivided into CD8 or CD4 positivity. The gates for the respective plots are: upper left: all cells; upper right: lymphocytes (FSC/SSC); lower left: lymphocytes (FSC/SSC); lower right: CD3+ lymphocytes. The relative values of parent gates are shown. The relative values of parent gates are shown.

Supplemental Table 2:

Course of Torque Teno Virus levels:

| **Date of measurement** | **TTV levels (copies/ml)** |
| --- | --- |
| 09.09.2015 | **4.5 x10^8^** |
| 22.01.2020 | **8.9 x10^4^** |
| 15.09.2020 | **1.9x10^5^** |
| 25.01.2021 | **Negative** |
| 26.04.2021 | **Negative** |
| 30.07.2021 | **Negative** |
